# Same admissions tools, different outcomes: a critical perspective on predictive validity in three undergraduate medical schools

**DOI:** 10.1186/1472-6920-13-173

**Published:** 2013-12-27

**Authors:** Daniel Edwards, Tim Friedman, Jacob Pearce

**Affiliations:** 1 Australian Council for Educational Research and Centre for Population and Urban Research, Monash University, Melbourne, Australia; 2 Australian Council for Educational Research, Melbourne, Australia; 3 Australian Council for Educational Research and the University of Melbourne, Melbourne, Australia

**Keywords:** Selection, Predictive validity, Admissions policy

## Abstract

**Background:**

Admission to medical school is one of the most highly competitive entry points in higher education. Considerable investment is made by universities to develop selection processes that aim to identify the most appropriate candidates for their medical programs. This paper explores data from three undergraduate medical schools to offer a critical perspective of predictive validity in medical admissions.

**Methods:**

This study examined 650 undergraduate medical students from three Australian universities as they progressed through the initial years of medical school (accounting for approximately 25 per cent of all commencing undergraduate medical students in Australia in 2006 and 2007). Admissions criteria (aptitude test score based on UMAT, school result and interview score) were correlated with GPA over four years of study. Standard regression of each of the three admissions variables on GPA, for each institution at each year level was also conducted.

**Results:**

Overall, the data found positive correlations between performance in medical school, school achievement and UMAT, but not interview. However, there were substantial differences between schools, across year levels, and within sections of UMAT exposed. Despite this, each admission variable was shown to add towards explaining course performance, net of other variables.

**Conclusion:**

The findings suggest the strength of multiple admissions tools in predicting outcomes of medical students. However, they also highlight the large differences in outcomes achieved by different schools, thus emphasising the pitfalls of generalising results from predictive validity studies without recognising the diverse ways in which they are designed and the variation in the institutional contexts in which they are administered. The assumption that high-positive correlations are desirable (or even expected) in these studies is also problematised.

## Background

Admission to medical school is one of the most highly competitive entry points in higher education. Considerable investment is made by universities to develop selection processes that aim to identify the most appropriate candidates for their medical programs. In this development a range of tools are utilised. The most common of these include previous academic achievement (usually indicated through school results), candidate interviews, references, personal statements and performance in an aptitude test [1]. In general, the aim of the selection processes developed by institutions is to identify individuals with the cognitive ability (or potential) to tackle the intellectual rigours of the course and non-cognitive traits (or potential) to negotiate the ethical, inter-relational, and motivational challenges of a medical degree.

The inevitable questions that are raised when developing or evaluating admissions processes are: How well do the tools and the process in its entirety predict the course performance of selected students? And, how well to these tools and processes identify individuals who will become good doctors? These questions of predictive validity are important, because they explore the justification for certain measures to be used. However, sometimes the importance of predictive power of admissions variables can be over-emphasised. Selection tools are usually designed to select who is likely to succeed, but not necessarily to predict actual performance in course assessment. In other words, they are used to identify who should be selected, rather than who is likely to perform the best. Further, and more fundamentally, the ability to succeed in a medical degree does not necessarily correlate with how good a medical doctor one will become.

This paper’s contribution is the critical perspective it adds relating to predictive validity in medical admissions (with an emphasis of course outcomes). It highlights the pitfalls of drawing generalised conclusions regarding selection tools without recognising the diverse ways in which they are designed and the variation in the institutional contexts in which they are administered. The paper also responds in part to Ferguson and colleagues [1] call for more work into the interaction and impact of multiple admissions tools. Additionally, it contributes to the small body of work specifically in this area in Australia and New Zealand.

### Background: admissions tools and predictive validity

There is a substantial body of research examining the predictive validity of admissions processes and in particular aptitude tests in medical education. A systematic review of more than 150 papers by Ferguson et al. [1] into the association between admissions variables and success in medical schools found that prior academic performance (measured through admissions tests or school results) was a moderate predictor of undergraduate achievement, accounting for up to 23 per cent of variance in medical school performance. While much evidence was found in Ferguson’s review relating to achievement variables as predictors of outcomes, the authors found less work undertaken on the role of interviews or personal statements and references. A key issue also highlighted by Ferguson was the need for more analyses into the combined impact of multiple admissions tools and the inter-correlation of such tools. New research in this area is not only appropriate but also timely, as the Ferguson et al. work was conducted over ten years ago.

In terms of specific tools for selection, the North-American based Medical College Admissions Test (MCAT) sets the standard worldwide in that it has a rich amount of research into its predictive validity with future performance in medical school. Meta-analyses of both Kyei-Blankson [2] and Donnon and colleagues [3] showed varying correlations between MCAT scores and future performance, with the former reporting correlations between the test and academic performance ranging between 0.10 and 0.50, and the latter reporting correlations between 0.39 and 0.60 between MCAT and licensing exam measures. Mixed results are also reported for UK based medical entrance examinations, the UK Clinical Aptitude Test (UKCAT) and the Biomedical Admissions Test [4-6].

The Undergraduate Health Sciences and Medical Admissions test (UMAT), one of the key tools of focus in this paper, is an aptitude test that has been used by Australian and New Zealand Universities since the early 1990s as part of the admissions process [7]. Twelve medical schools in Australia and New Zealand currently use UMAT, while similar versions of this instrument are also administered in Ireland by medical schools to assist in selection of applicants to undergraduate medical degrees.

The specific exploration of predictive validity of UMAT alongside other admissions tools has been considered in more detail through a number of studies in more recent years. Wilkinson and colleagues [8] reported a weak correlation between UMAT and undergraduate grade point average scores in a study relating to the University of Queensland across four years (two years in a prior degree and the first two years of medical studies), which led the authors to concluded that UMAT has limited predictive validity as a medical selection tool. However, the nature of this conclusion was challenged by Griffin [9].

A study by Mercer and Puddey [10] exploring 11 cohorts in the medical program at the University of Western Australia found no relationship between the total UMAT score, and the weighted average mark achieved by students. However, some correlations were found with individual components of UMAT and specific years of study. The authors found that school outcomes independently predicted GPA consistently through the course, although correlations reduced in later years of study. At this institution the interview was found to be an independent predictor in the clinical years rather than in the early years of the degree.

The only previous multi-institution investigation of the predictive validity of UMAT, conducted across two New Zealand universities reported that UMAT scores used in combination with admission GPA had a small but positive contribution in predicting outcomes on all major summative assessments from years 2 to year 6 of the undergraduate programs in comparison to just using admissions GPA alone [11]. The authors noted that the relationship between UMAT and outcomes measures was stronger in later years. The pattern of the relationship between UMAT and outcomes variables was similar across both institutions.

In the context of previous studies of medical admissions, it is pertinent to explore the extent to which predictive validity should be a marker of success for institutions. Do the diverse ways in which admissions tools are designed and administered in a variety of institutional contexts influence the outcomes achieved by medical schools? Further, are these multiple variables a strength or a weakness of admission processes? And importantly, do they highlight any pitfalls regarding the tendency to generalise results from studies into predictive validity?

## Method

### Population

This study examines 650 undergraduate medical students from three Australian universities as they progressed through the initial years of medical school. The three (de-identified) institutions involved are all members of the UMAT Consortium, a group of universities who own, and oversee the development and administration of UMAT. Each institution involved volunteered to be part of this research and provided the data relevant for the study. The work was funded by the UMAT Consortium through a competitive research grant. All data was provided to the researchers by participating universities, individual data was de-identified prior to provision. The work was carried out in accordance to the Australian Council for Educational Research Code of Ethics, project number 233423.

Two cohorts of students were used, those who sat UMAT in 2005 and were successful in being admitted to university in 2006 (n = 224) and those who took UMAT in 2006 and were successful for entry in 2007 (n = 426). Data varied across the institutions with regards to cohort participation and years of GPA data available (Table 1). The number of students who withdrew from their course of study at some stage during the period of data collection was less than six per cent. In total, the students included in this study accounted for approximately 25 per cent of all commencing undergraduate medical students in Australia in 2006 and 2007.

**Table 1:** Number of students per cohort and data availability and range of GPA for each institution

**Institution**	**GPA data availability**	**GPA range**	**Number of students in 2006 cohort**	**Number of students in 2007 cohort**
**A**	Years 1, 2, 3	1 to 8	0	102
**B**	Years 1, 2, 3, 4	0 to 100	172	229
**C**	Years 1, 2	0 to 100	52	95

### Admissions and outcome variables

Australian medical schools generally use a combination of three components for selection to undergraduate medical degrees: an academic score (generally based on school achievement), UMAT, and an interview. Each institution in this study used different combinations of these admissions variables. For example, two of the institutions used interview, of which one was based on Multiple-Mini Interview (MMI) and the other a semi-structured interview approach with a three-member interview panel, while one institution only used school outcome and UMAT, with no interview.

The UMAT consists of three sections: Section 1 – Logical Reasoning & Problem Solving Section 2 – Understanding People; and Section 3 – Non-Verbal Reasoning. The three sections can be combined to form a Total score. UMAT is used in different ways by the institutions in this study, varying in the use of section/total scores and the weighting in the admissions decision making process.

The measure of school performance (School) is based on an aggregation of subject scores undertaken in the final year of secondary schooling and then applied to a percentile ranking across the age cohort [12]. Each institution used a different but equivalent state-based ranking.

**Figure 1:**
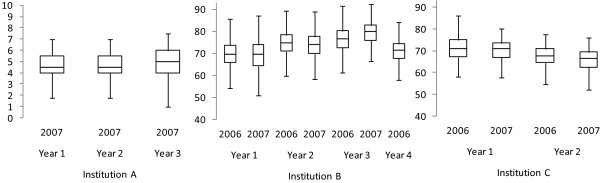
GPA distribution across institution, cohort and year of study.

The outcome variable analysed here is Grade Point Average (GPA) data from each of the first four years of study (first three years for students in the 2007 cohort). There was no GPA data available for the third and fourth years of study at Institution C because students were assessed as pass/fail, without any quantitative grade made available. The GPA distributions varied across institutions (Figure 1). No significant differences were found between cohorts of students for the different year levels at each institution (based on Mann Whitney U tests), with the exception of Year 3 at Institution B. Because of the similarity of distributions across cohorts, the analyses in this report use combined GPA data from both the 2006 and 2007 cohorts. The number of students who withdrew from their course of study at some stage during the period of data collection was less than six percent.

To limit problems that may arise during statistical estimation, GPA data were transformed onto a z-score distribution with a mean of zero and standard deviation of one. Data were standardised within years of performance, within cohorts, and within institutions. Even given this standard metric, however, it is important to stress that different institutions’ GPAs are not equated. That is, a GPA of 1.5 at Institution A is likely to reflect a different standard of student achievement than a GPA of 1.5 at Institution B. In addition, while all medical courses are accredited and regularly audited, the quality and performance of the criterion variables are unknown. Unfortunately, due to the lack of moderation or calibration processes in Australian higher education, it was not feasible to psychometrically equate student assessment data and combine GPAs into a common variable.

### Analyses

All analyses were conducted using IBM SPSS Statistics Version 19. Pearsons r correlations were performed between admissions variables and GPAs across institutions, entrance cohorts and year levels. The authors then used standard multiple regression (ordinary least squared) modelling in order to isolate the role that each of the admissions variables plays in influencing the achievement of students in the first three to four years of the degree. These analyses were run for all institutions across years and entrance cohorts. Treatment of a UMAT score for restriction of range was carried out in accordance with the formulae and methodology applied in numerous studies of predictive validity [5,10,13]. No form of adjustment for multiple comparisons (Type 1 error) were made to the reported data.

## Results

Correlations between GPA and admissions variables (adjusted UMAT section and total scores, school performance, interview) for each institution are presented in Table 2. Academic performance in the first two year-levels was found to correlate with the UMAT Total score, with the exception of Year 2 at Institution C. A significant correlation was reported for Year 3 at Institution A, but not for Years 3 or 4 at Institution B.

**Table 2:** Pearsons correlations of admissions variables and course GPA by institution and year level

**Institution**	**Level**	**UMAT**				**Interview**	**School**
		**Logical reasoning**^**#**^	**Understanding people**^**#**^	**Non-verbal reasoning**^**#**^	**Total**^**#**^		
**A**	Year 1	**0.40**	0.21	−0.06	**0.34**	**−0.31**	**0.26**
	Year 2	**0.47**	**0.43**	−0.14	**0.41**	**−0.39**	0.19
	Year 3	**0.48**	**0.47**	−0.18	**0.41**	0.05	**0.21**
**B**	Year 1	**0.18**	**0.29**	−0.01	**0.26**	0.04	**0.41**
	Year 2	**0.16**	**0.19**	−0.01	**0.19**	0.06	**0.39**
	Year 3	−0.03	0.05	−0.05	−0.03	**0.12**	**0.23**
	Year 4	0.08	**0.17**	0.00	0.15	0.08	**0.28**
**C**	Year 1	**0.26**	0.18	0.14	**0.32**		0.12
	Year 2	0.16	−0.09	**0.18**	0.14		0.19

Bolded coefficients *p* < 0.05.

^#^adjusted for restriction of range.

Exploring the individual section scores of UMAT, the correlations in the first two years appears to be largely driven by the Logical Reasoning and Understanding People sections. Logical Reasoning correlated strongly at all institutions within the first two year-levels. Understanding People correlated with performance in Year 1 in Institution B and had stronger correlations in Years 2 and 3 at Institution A. There was less of a relationship between academic performance and Non-verbal Reasoning scores, with only the correlation at Year 2 for Institution C reaching significance.

The relationship between interview scores and academic performance was largely insignificant or negatively correlated across institutions. An exception being the correlation for Year 3 at Institution B. School performance was the strongest at Institution B with significant correlations across all four years. In Institution A, school performance was significant in Year 1 and Year 3 but the UMAT total scores recorded larger correlations. For Institution C there was no significant correlation between school performance and GPA, while for UMAT, the only significant correlation was with the total UMAT score and first year GPA.

Table 3 presents the results from linear regression models that regress each of the three admissions variables on GPA, for each institution at each year level. The regression models incorporate the three UMAT section scores as opposed to the total score.

**Table 3:** Standardised regression coefficients (p-values in brackets) and explained variance of regression models by institution and year level

**Institution**	**Level**	**UMAT**						**School**		**Interview**		**R**^**2 **^**of model**
		**Logical reasoning**		**Understanding people**		**Non-verbal reasoning**						
**A**	Year 1	0.21	(0.03)	0.17	(0.09)	−0.06	(0.52)	0.25	(0.01)	−0.29	(0.00)	0.23
	Year 2	0.25	(0.01)	0.25	(0.01)	−0.19	(0.05)	0.15	(0.10)	−0.44	(0.09)	0.36
	Year 3	0.24	(0.02)	0.25	(0.01)	−0.16	(0.14)	0.15	(0.18)	0.03	(0.76)	0.16
**B**	Year 1	0.02	(0.63)	0.14	(0.00)	−0.14	(0.00)	0.44	(0.00)	0.03	(0.45)	0.21
	Year 2	0.01	(0.84)	0.07	(0.12)	−0.13	(0.01)	0.42	(0.00)	0.04	(0.35)	0.18
	Year 3	0.08	(0.15)	0.00	(0.99)	−0.11	(0.04)	0.28	(0.00)	0.11	(0.04)	0.17
	Year 4	−0.02	(0.80)	0.08	(0.33)	−0.09	(0.32)	0.33	(0.00)	0.12	(0.14)	0.11
**C**	Year 1	0.08	(0.45)	0.03	(0.76)	0.05	(0.67)	0.10	(0.36)	N/A		0.03
	Year 2	0.13	(0.25)	−0.07	(0.55)	0.03	(0.78)	0.16	(0.14)	N/A		0.06

The pattern of significance of regression coefficients is shown to vary considerably across years of progression and across institutions. At Institution A, all 5 admissions measures were found to be significant (at the 0.05 level) in at least one of the three years, although only Logical Reasoning was significant across all three and Interview was negative. At Institution B, school performance was significant across all four year levels, while Non-verbal Reasoning was significant for three years, although notably with negative regression coefficients. At Institution C, no regression coefficient was significant.

The combined explanatory power (R^2^) of the admissions variables is also shown in Table 3. The highest result here can be seen for Institution A Year 2, where these admissions tools combine to explain 36 per cent of the variance in GPA. In Institution B the strength of the explanatory power declines at each year level, but is still 0.17 (or 17 per cent) at Year 3. In contrast to the other two institutions, the combined admissions tools offer very little explanatory power in Institution C.

**Figure 2:**
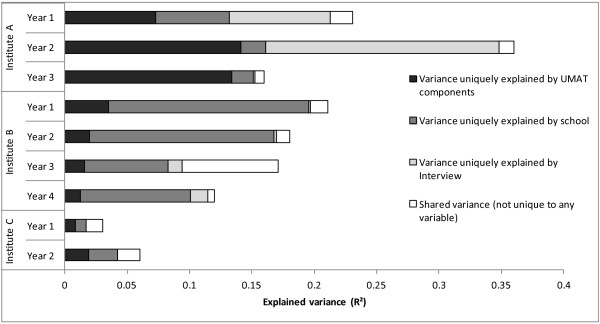
Relative contribution to explained variance of each admissions variable, and variance shared by all variables.

The contribution of each admission variable to the explained variance is displayed in Figure 2. Squared semi-partial regression coefficients indicate how much of the R^2^ can be accounted for uniquely by each admissions variable. The figure shows a differential pattern of relative contribution to explained variance by the three admissions variables, according to both institution and year level. At institution A, the vast majority of explained variance in Years 1 and 2 is explained by contributions from both UMAT components and Interview scores, although the association between the latter and GPA appears to be negative. In Year 3 the vast majority of explained variance is accounted for solely by UMAT. At Institution B, school performance contributes the most to the explained variance of the admission variables across the four levels. The models account for only a small amount of variance at the two year levels of Institution C. Importantly, this figure shows that each of the admissions tools used contributes to explaining the variance in outcomes independent of the influence of other tools. Essentially, this shows incremental validity.

## Discussion

Overall, the data in this analysis has shown positive correlations between performance in medical school, school achievement and UMAT, but not interview. However, there were substantial differences between schools, across year levels, and within sections of UMAT exposed in particular through the regression analyses. Despite this, each admission variable was shown to add towards explaining course performance, net of other variables.

### UMAT

While UMAT is designed as a selection tool rather than a predictor of outcomes, any evidence to suggest that it assists as a predictor of university outcomes is an additional benefit of the test. Overall there were mixed results across the three schools involved. In Institution A UMAT was shown to correlate strongly with Total score of 0.41 with 2^nd^ and 3^rd^ Year results and up to 0.48 with Logical Reasoning in Year 3. These levels of correlation were not as strong in the other institutions although in general for GPAs at Year 1 and Year 2 UMAT was shown to predict scores within the same range as identified for MCAT and UKCAT [1-4,6]. When the role of UMAT is examined net of the influence of the other admissions tools it was shown to play an important role in explaining the variance in medical school outcomes in two of the three institutions in this study. For Institution C none of the admissions measures were particularly good predictors of GPA.

An argument can be made that a low, positive correlation between UMAT and medical school performance is desirable. UMAT is designed to be highly discriminating; the test is deliberately selected to maximise discrimination around putative selection thresholds, thus forgoing fine discrimination amongst those who clearly exceed this threshold. Course assessments rarely have a large number of assessment items to discriminate at the high end, and are largely content based, especially in the early years. A high-positive correlation is thus not expected, as the instruments are designed for differing purposes. The low, negative correlation with Non-verbal Reasoning is also understandable. Non-verbal Reasoning targets a very different skill to what is commonly assessed in university outcomes. The different parts of UMAT are designed to assess different aspects of cognitive ability―aspects which are unlikely to produce strong correlations with aggregated GPAs. Different parts of UMAT may correlate more highly with different individual assessments at university. Studies to investigate correlations between practical components of undergraduate medicine and Understanding People section of UMAT, for instance, would be valuable.

### School achievement

As consistently found in studies of predictive validity [1,10,14], school achievement generally recorded the strongest correlations of the three measures used by the institutions in this study. In particular school achievement was strongly correlated with GPA in Institution B, with a correlation of 0.41 in Year 1 and 0.39 in Year 2. This is as expected. School examinations and early year medical school examinations are not only both content based, they are both instruments which produce weighted results.

### Interview

Of the two institutions in this study which use an interview, the predictive power identified appears to be due to a negative association with GPA (indicating poorer performance on the interview is associated with greater performance in GPA). This is not necessarily a poor reflection on the interview as in general this tool is not about identifying high or low performing students, but rather focussing on non-cognitive traits, so as a predictor of GPA it would not necessarily be expected to perform strongly. This is a clear case where a strong, positive correlation is not necessarily desirable, nor should it be anticipated.

### Admissions measures as a combined tool

While there are differences revealed between institutions, across years and among the tools used for admissions, one constant shown through this study is that the use of multiple tools does result in an increase in predictive validity. For all three institutions, each selection tool incrementally added to the explaining the variance of outcomes of medical school. It should be noted however, that the three variables were only able to explain between 3 and 36 per cent of variance in GPA, indicating that these admissions variables only explain a relatively small proportion on how students will perform in their studies.

### Limitations

While there are interesting outcomes in the analyses in this study, the key lesson from this work is that even within a study that attempts to standardise processes and data for comparison, there is massive variation in results across institutions for each of the main admissions tools used in undergraduate medical selection. Standardising the variables to ensure valid comparison is not straightforward. There are a range of issues that are no doubt influencing these outcomes, all of which are important for monitoring in future work.

One influence on the results shown in this predictive validity study is the variability in admissions processes across universities [14]. While all three institutions used school achievement scores and UMAT, and two of the three used interview, the application of these tools into the process of selection is different at each of these universities and across all schools in Australia [10].

In addition, the criterion variables in predictive validity studies—the within course assessments on which GPA is based—also vary across institutions [8]. Not only are GPAs calculated differently among universities, but more importantly, the assessments on which the marks for the GPAs are derived differ substantially. While largely unknown, it is likely that there are also differing levels of validity and reliability across the range of these assessments. The diversity in institutional variables, coupled with the varying rationales in the specific design of the different selection tools must not be underestimated.

## Conclusion

The findings here suggest the strength of multiple admissions tools in predicting outcomes of medical students. However, more importantly, the paper highlights the issues that underscore the complex context around which predictive validity studies are instigated. The assumption that high-positive correlations are good or even expected in these studies requires critical reflection. The findings here therefore offer an additional insight into analyses of predictive validity, while also providing some perspective on the difficulty in deriving accuracy and generalisability from such research.

## Competing interests

The authors of this paper are employed by the Australian Council for Educational Research, which develops and administers UMAT for medical schools in Australia and New Zealand. This study was funded through a competitive research grant provided by the UMAT Consortium, the group of medical schools which own and oversee UMAT.

## Authors’ contributions

DE led this research project and was responsible for writing the manuscript. TF ran the analyses for the research and contributed substantially to the development of the manuscript. JP was closely involved in the development of the manuscript. All authors read and approved the final manuscript.

## Pre-publication history

The pre-publication history for this paper can be accessed here:

http://www.biomedcentral.com/1472-6920/13/173/prepub
